# Religiosity and teen birth rate in the United States

**DOI:** 10.1186/1742-4755-6-14

**Published:** 2009-09-17

**Authors:** Joseph M Strayhorn, Jillian C Strayhorn

**Affiliations:** 1 Drexel University College of Medicine, Department of Psychiatry, 2900 W. Queen Lane, Philadelphia, PA 19129, USA; 2 University of Pittsburgh School of Medicine, Department of Psychiatry, 3811 O'Hara Street, Pittsburgh, PA 15213, USA; 3 263 Seasons Drive, Wexford, Pennsylvania 15090, USA

## Abstract

**Background:**

The children of teen mothers have been reported to have higher rates of several unfavorable mental health outcomes. Past research suggests several possible mechanisms for an association between religiosity and teen birth rate in communities.

**Methods:**

The present study compiled publicly accessible data on birth rates, conservative religious beliefs, income, and abortion rates in the U.S., aggregated at the state level. Data on teen birth rates and abortion originated from the Center for Disease Control; on income, from the U.S. Bureau of the Census, and on religious beliefs, from the U.S. Religious Landscape Survey carried out by the Pew Forum on Religion and Public Life. We computed correlations and partial correlations.

**Results:**

Increased religiosity in residents of states in the U.S. strongly predicted a higher teen birth rate, with r = 0.73 (p < 0.0005). Religiosity correlated negatively with median household income, with r = -0.66, and income correlated negatively with teen birth rate, with r = -0.63. But the correlation between religiosity and teen birth rate remained highly significant when income was controlled for via partial correlation: the partial correlation between religiosity and teen birth rate, controlling for income, was 0.53 (p < 0.0005). Abortion rate correlated negatively with religiosity, with r = -0.45, p = 0.002. However, the partial correlation between teen birth rate and religiosity remained high and significant when controlling for abortion rate (partial correlation = 0.68, p < 0.0005) and when controlling for both abortion rate and income (partial correlation = 0.54, p = 0.001).

**Conclusion:**

With data aggregated at the state level, conservative religious beliefs strongly predict U.S. teen birth rates, in a relationship that does not appear to be the result of confounding by income or abortion rates. One possible explanation for this relationship is that teens in more religious communities may be less likely to use contraception.

## Background

The children of teen mothers in the U.S., on the average, have worse outcomes in a number of ways. They score lower in school achievement tests, have a greater likelihood of repeating a grade, are rated more unfavorably by teachers while in high school, have worse physical health, are more likely to be indicated victims of abuse and neglect, have higher durations of foster care placement, and are almost three times more likely to be incarcerated during adolescence or the early 20 s than the children of mothers who delayed childbearing; the daughters of teen mothers are more likely to become teen mothers themselves[1].

In the United States, what to teach adolescents about sexuality and the prevention of teen pregnancy has been controversial. A number of sex education programs in the U.S. have been mandated to be "abstinence-only" programs, excluding the teaching of contraceptive techniques. As stated in a National Public Radio poll report, "the historical impetus for abstinence education has come from evangelical or born-again Christians.... Eighty-one percent of evangelical or born-again Christians believe it is morally wrong for unmarried adults to engage in sexual intercourse, compared with 33 percent of other Americans....More than twice as many evangelicals as non-evangelicals (49 percent to 21 percent) believe the government should fund abstinence-only programs instead of using the money for more comprehensive sex education [2]."

Other polls have presented varying results on similar questions: A 2008 poll in Minnesota [3] reported that a significantly smaller fraction of those who described themselves as "very conservative" politically and those who were "born again" Christian supported comprehensive sex education than the corresponding fractions of more liberal and non-born-again; however, in this sample, 83.2% of the born-again Christians supported comprehensive sex education; only 51% of the politically "very conservative" supported it.

The connection between religion and attitudes toward contraception prompts investigation of the relationship between religiosity and teen pregnancy.

Some studies have suggested that greater religiosity is associated with either greater abstinence or lower teen birth rate. Hardy and Raffaelli, who analyzed data from the National Longitudinal Survey of Youth, reported that higher time one religiosity predicted a lower likelihood of first sexual intercourse between time one and time two [4]. Loury concluded that communities with larger communities of Catholics and Conservative Protestants have lower rates of teen childbearing, all other things equal [5]. This conclusion was drawn from an analysis of women from age 14-20 in 1979, taken from the National Longitudinal Study of Youth. McCree and colleagues found that African-American females with higher religiosity scores were more likely to have initiated sex at a later age, to have used a condom in the last six months, and to possess more positive attitudes toward condom use [6]. Rostosky et al. found that adolescent religiosity predicted later coital debut [7]. However, there was a significant interaction between race and religiosity: African-American adolescent males who were either more religious or had signed a virginity pledge were more likely to debut than African-American males who were less religious and/or who had not signed a pledge. Miller and Gur found, upon analyzing the National Longitudinal Study of Adolescent Health in the U.S., that frequent attendance of religious events in girls 12 to 21 years old was positively associated with a "responsible and planned use of birth control" [8]. Personal conservatism, however, was associated with unprotected sex. Manlove and colleagues, upon analysis of the 1997 National Longitudinal Survey of Youth, found that in the sample as a whole, greater family religiosity was associated with "using contraceptives consistently"; however, among sexually active males, family religiosity was "directly and negatively associated with contraceptive consistency" [9].

Other studies have suggested that religiosity is associated with behaviors that could lead to a higher teen birth rate. Studer and Thornton found that among 18 year-olds, religious teenagers were less likely to use medical methods of contraception when sexually active [10]. Dodge and colleagues compared male college students in the United States and the Netherlands [11]. American men reported higher rates of inadequate contraception and unwanted pregnancy than their Dutch counterparts; religiosity and sex education were thought to explain these differences.

Rosenbaum compared adolescents who reported taking a virginity pledge with a matched sample of nonpledgers [12]. Among the matching variables was pre-pledge religiosity and attitudes toward sex and birth control. Pledgers did not differ from nonpledgers in lifetime sexual partners and age of first sex, but pledgers were less likely to have used birth control and condoms in the past year and at last sex. This research raises the possibility that moralistic attitudes toward sexuality can actually increase the likelihood of pregnancy, by discouraging contraception without successfully discouraging sexual intercourse.

Such a hypothesis is bolstered by the research of Santelli and colleagues, who calculated that 86% of the decline in adolescent pregnancies that occurred between 1995 and 2002 was attributable to improved contraceptive use [13]. Santelli and colleagues cite the example of the Netherlands, which in the 1970's went through a period of soul searching and consensus-building about the need for contraception and prevention of sexually transmitted infections in adolescents, and today has one of the lowest teen birth rates in the world[14]. If contraception is more effective than attempted abstinence in reducing birth rates, then attempts to discourage both contraception and sexual intercourse among teenagers could raise teen birth rates.

A complicating variable related to teen births and religiosity is the rate of abortions among teens. Adamczyk and Felson, after analyzing longitudinal survey data from the U.S., reported that more highly religious women are less likely to have either an abortion or an out of wedlock pregnancy [15]. Tomal, upon analyzing data from 1024 counties in 18 U.S. states, found that religious membership level was negatively related to teen abortion rates [16].

Cahn and Carbone summarized differences in attitudes about family and sexuality between the more religious and conservative U.S. "red families," versus the less religious and more liberal "blue families" [17]. These authors observed: "Within red families, abstinence outside of marriage is a moral imperative, the shotgun marriage is the preferred solution to an improvident pregnancy, and socialization into traditional gender roles is critical to marital stability." The blue model, however, "involves less control of sexuality, celebrates more egalitarian gender roles, and promotes financial independence and emotional maturity as the sine qua non of responsible parenthood In this new model, abstinence is unrealistic, contraception is not only permissible, but morally compelled, and abortion is the necessary (and responsible) fallback." (p. 3). Cahn and Carbone mention that teen birth rates appear higher among "red" families.

The present study approaches the relationship between teen birth rate and religiosity by looking at data aggregated across states in the United States.

## Methods

### Data Sources

This study compiled data from publicly accessible data sets. The data on religiosity were from the U.S. Religious Landscapes Survey, published by the Pew Forum on Religion and Public Life in 2008 [18]. The Pew survey was conducted in 2007, with additional subjects added in 2008; it employed telephone survey methodology with a sample of 35,957 participants. We used the results of eight questions from the survey, the responses to which were broken down by state. We transcribed the percent of respondents who endorsed the most conservative religious answer to each of the eight questions. Specifically, we entered the percentages of respondents for each state who endorsed each of the following statements:

1) Belief in a God or universal spirit: Absolutely Certain.

2) There is only one way to interpret the teachings of my religion.

3) Scripture should be taken literally, word for word.

4) How important is religion in your life: Very Important.

5) My religion is the one true faith leading to eternal life.

6) Frequency of attendance at religious services: at least once a week.

7) Frequency of prayer: at least once a day.

8) How often do you receive a definite answer to a specific prayer request: at least once a month.

In the tables published in the Religious Landscapes Survey, the percents reported were aggregated across three pairs of states and across Maryland and the District of Columbia, because the sample size from at least one member those pairs was fairly small. We obtained from the Pew Forum staff the disaggregated data and used those numbers in our data set. The sample sizes were deemed too small in Wyoming and the District of Columbia for Pew Forum to release them, and thus these data points are missing. For Rhode Island, data were missing on two of the eight questions; we imputed these missing data points by means of regression on the remaining six questions, so that Rhode Island could be included in the data set.

In order to obtain one composite religiosity score for each state, we averaged the percents of respondents endorsing the most religious answer across the eight questions.

The rates of teen birth in the fifty U.S. states plus the District of Columbia were reported by the National Center for Health Statistics at the Centers for Disease Control and Prevention [19]. The data reported were for 2006 births, the latest available (and thus the closest possible in time to the date of the collection of the religiosity data set).

A possible confounding variable in the relationship between teen birthrate and religiosity is household income level. We obtained data on the median household income by state in the U.S. from data published by the U.S. Census Bureau [20]. The median two year average household income for 2006-2007 for each state was entered into our database.

To account for another factor which could complicate the analysis of teen birthrate and religiosity, we estimated the abortion rate among teenagers for each state. The most recent data available on abortion rates were from 2005, published by the Center for Disease Control [21]. These rates were broken down by the states of residence of the women receiving the abortions. In order to estimate rates for abortions delivered to teens only, we multiplied the overall rates by the fraction of abortions delivered to teens for 2005, as published in the same Centers for Disease Control report; these were categorized by the state in which the abortion was delivered. Data were available for 46 states; the District of Columbia. California, Florida, Louisiana and New Hampshire were missing from this data set. The product of the abortion rate and the fraction of abortions delivered to teens yielded an estimated rate of abortions per 1000 teenaged females.

The CDC obtains its abortion rates by surveying the Central Health Departments of the various regions. A different approach is used by the Guttmacher Institute, which surveys providers of abortions. We used the Guttmacher data for 2005 to cross check abortion rates [22].

### Data Analyses

We examined the intercorrelations among the individual religiosity questions to determine whether these were high enough to form an index score. We then formed an index score by averaging the eight religiosity items.

We examined the relationships of the variables with Pearson correlations and partial correlations, as computed by SPSS [23]. The partial correlation between a first and second variable, controlling for the third, is identical to the Pearson correlation between the residuals obtained when each of the first two is regressed upon the third - in other words, when the effect of the third variable is "removed" from each of the first two [24]. We computed 95% confidence intervals for the most important correlations and partial correlations, using the Fisher r-to-z transformation. The variance of the Fisher-transformed correlation is 1/(n-3) for bivariate correlations, and 1/(n-k-2) for partial correlations, where k is the total number of independent variables (e.g. k = 2 for a partial correlation with one variable controlled; k = 3 for two variables controlled) [24].

## Results

### The justification for forming an index from the Pew religion items

We examined the 28 intercorrelations among the eight different religiosity variables reported in the Pew Survey. The minimum intercorrelation was 0.55, and the maximum was 0.96. The average intercorrelation was 0.81. Thus the intercorrelation of the religiosity items are high enough to justify making an index score by averaging the scores across the eight items.

### The correlation between teen birth rate and religiosity

For all the correlational analyses reported below, we examined the plots of residuals for the regressions with the same independent variables and with teen birth as the dependent variable. There was a slight trend toward increasing residuals with increasing values of the dependent variable; in our opinion this trend was not enough to invalidate the linear model, in view of the high correlations obtained and the linear appearance of scatter plots.

**Figure 1:**
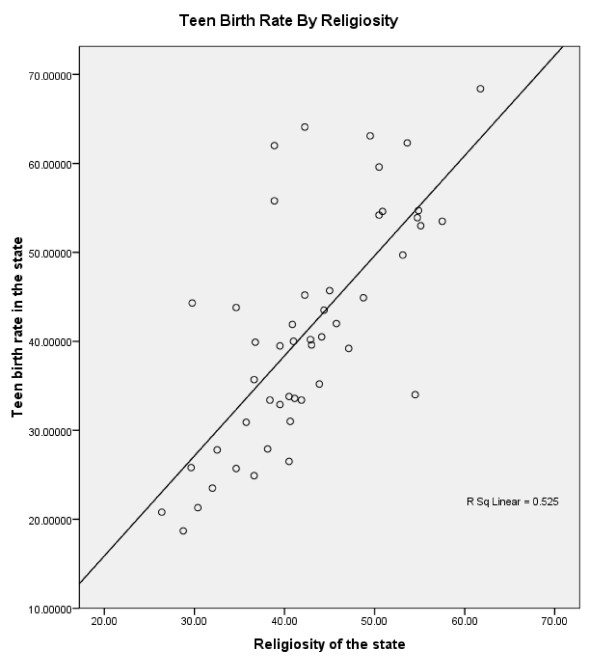
**Scatterplot of Teen Birth Rate by Religiosity Score**.

Teen birth rate correlated with our composite religiosity variable with r = 0.73; 95% CI (0.56,0.84); n = 49; p < 0.0005. Thus teen birth rate is very highly correlated with religiosity at the state level, with more religious states having a higher rate of teen birth. A scatter plot of teen birth rate as a function of religiosity is presented in Figure 1.

### Controlling for income and abortion rate

Next we considered whether median family income for states could be a confounding variable. Income negatively correlated with teen birth rate, with r = -0.63, n = 51, p < 0.0005. Furthermore, income correlated negatively with religiosity, with r = -0.66, n = 49; p < 0.0005. Thus the direction and magnitude of correlations made income a primary candidate for a confounding variable. However, the partial correlation of teen birth rate with religion while controlling for income was 0.53; 95% CI (0.29, 0.71); df = 46; p < 0.0005. Thus the correlation between religion and teen birth remained large and highly significant, even when controlling for income. The raw religiosity scores and teen birth scores shared a little over half their variance (R^2 ^= 0.53) whereas these variables with income removed by partialing shared a little over a quarter of their variance (R^2 ^= 0.28).

The correlation between teen abortion rate and religiosity was -0.45; n = 45; p = 0.002. Thus the teen abortion rate was lower in states that were more religious. Furthermore, teen abortion rate was negatively correlated with teen birth rate, with r = -0.26, although this relationship failed significance at the 0.05 level (n = 47, p = 0.078). Would including abortion rate as a covariate greatly affect the correlation between teen birth and religiosity? The answer turned out to be no. The partial correlation between teen birth rate and religiosity, controlling for abortion rate, was 0.68; 95% CI (0.48, 0.81); df = 42; p < 0.0005. The partial correlation between teen birth and religiosity, controlling for both income and abortion rate, was 0.54; 95% CI (0.29, 0.72); df = 41; p < 0.0005. Thus, even after taking into account the abortion rate and controlling for income, the correlation between religiosity and teen pregnancy remained high and significant.

Table 1 presents a summary of the four correlations that summarize our findings on the relationship between teen birth and religiosity.

**Table 1:** Correlation or Partial Correlation of Teen Birth Rate with Religiosity with Various Variables Controlled

**Variable(s) Controlled**	**None**	**Income**	**Abortion**	**Income and Abortion**
Correlation or Partial Correlation	0.73	0.53	0.68	0.54

### Checks for Robustness

When averaging the results of the eight items of the religiosity survey, one approach would be to first compute the z-scores for each item, and then average the z-scores. Such an approach would assign the same standard deviation to each item, so that items with higher standard deviations did not count more heavily toward the average score. When we checked this "average of standardized religiosity scores," its correlation with the average of the raw percents was 0.999 (n = 49, p < 0.0005). This result implies that the two measures are interchangeable, and that differences in standard deviations among the eight items do not appreciably influence the distribution of the average religiosity score. We used the raw percents rather than the standardized scores so that the scatterplot would be more intuitively interpretable.

Was there something about the averaging process itself that hid important information or inflated the correlation? To check this, we computed the individual correlation with teen birth rate for each of the eight religiosity items. The results are presented in Table 2.

**Table 2:** Correlations of Teen Birth Rate with Individual Religiosity Items

**Item**	**Belief God**	**One Interp.**	**Literal Scripture**	**Import. For Life**	**One True Faith**	**Attend Services**	**Frequent Prayer**	**Prayers Answered**
Correlation	0.67	0.68	0.72	0.70	0.56	0.53	0.71	0.74

Averaging of items probably results in a higher reliability, which would be expected to improve the correlation with teen birth; the higher reliability of the average than of the individual items is predicted by the Spearman-Brown formula [25]. However, each of the items separately reveals a reasonably high correlation with teen birth rate.

We used as the measure of religiosity the percent of respondents replying in the most religious way; how would the conclusion have been affected if we had entered the percent replying in the least religious way? Some of the questions were dichotomies, and thus the correlations for those items would have had the same magnitude with opposite sign. To check a couple of the items that were not dichotomies, we entered the percentages for the least religious response to literal interpretation of scripture and frequency of prayer, and found correlations of -0.91 and -0.90, respectively, between percent most religious and percent least religious. The correlations of teen birth with "irreligiosity" were very similar in absolute value to the correlations with religiosity for those items. The percent who prayed "seldom or never" in the states correlated at -0.67 with teen birth; the percent who felt that scripture was a "book written by men, not the word of God" correlated at -0.63 with teen birth. It appears that alternate scoring mechanisms measuring irreligiosity would yield the same conclusions and would add nothing to our results.

The Guttmacher Institute gathers data on abortion rates by contacting providers of abortions rather than central health agencies. We entered into our data set the Guttmacher abortion rate for women 15-44 for 2005; the correlation of Guttmacher abortion rate (all women) with CDC rate is 0.66 (n = 47, p < 0.005). Using the Guttmacher abortion rate rather than the CDC rate in our partial correlations made no substantive changes - for example, the partial correlation of teen birth with religiosity controlling for CDC estimated teen abortion rates was 0.68; the partial correlation controlling for Guttmacher estimated teen abortion rates (obtained by multiplying overall Guttmacher abortion rates by the fraction of abortions obtained by teens according to CDC data) was 0.65.

The variation among the fraction of teen abortion rates in different states was small enough that it made little difference for the partial correlations whether we used estimated teen abortion rates or the overall abortion rates for women in the state. The partial correlation of teen birth with religiosity, partialling out CDC abortion rates for all women was 0.69; the same partial correlation using overall rates reported by Guttmacher was 0.70. Estimated teen abortions correlated with overall abortions for the state with r = 0.97 for CDC rates and r = 0.99 for Guttmacher rates.

To what extent is the main finding reported here, i.e. the correlation between teen birth and religiosity, dependent upon any one state? Inspection of the scatterplot reveals no major outliers; two influential points appear to be those for Mississippi and Utah. Mississippi, as the state both highest in religiosity and teen birth, tends to increase the correlation; however if the correlation is recomputed without Mississippi, the correlation remains in the same region, with r = 0.69. Utah, which is high in religiosity but in the mid range for teen birth, tends to decrease the correlation; with Utah (but not Mississippi) eliminated the correlation between teen birth rate and religiosity would have been 0.76. For Rhode Island, we used imputation to estimate the two out of eight items that were missing; had we simply made Rhode Island a missing data point, the correlation between teen birth and religiosity would have been 0.72. Thus the magnitude of the correlation we report appears not to be greatly altered by the elimination of any one state.

## Discussion

At the state level in the U.S., religiosity, as operationally defined by the eight questions of the Pew Survey, accurately predicts a high teen birth rate. The significant and high correlation continues to hold after statistically controlling for income and abortion rate.

It is a statistical maxim that higher correlations are to be found using aggregated data, for example state averages, than with individual level data. This is because some of the noise at the individual level is cancelled by the aggregation process, allowing the relationship between signals to be more clear. As stated in an introductory statistics text, "Correlations based on averages are usually too high when applied to individuals[26]." Nonetheless, the magnitude of the correlation between religiosity and teen birth rate astonished us. Teen birth is more highly correlated with some of the religiosity items than some of those items are correlated with each other. We would like to emphasize that we are not attempting to use associations between teen birth rate and religiosity, using data aggregated at the state level, to make inferences at the individual level. It would be a statistical and logical error to infer from our results, "Religious teens get pregnant more often." Such an inference would be an example of the ecological fallacy, which was explicated by Robinson in 1950 [27] and reviewed by Freedman in 2001 [28]. The associations we report could still be obtained if, hypothetically, religiosity in communities had an effect of discouraging contraceptive use in the whole community, including the nonreligious teens there, and only the nonreligious teens became pregnant. Or, to create a different imaginary scenario, the results could be obtained if religious parents discouraged contraceptive use in their children, but only nonreligious offspring of such religious parents got pregnant. We create these scenarios simply to illustrate that our ecological correlations do not permit statements about individuals.

We should also caution that on an individual level, certain teen pregnancies are often highly desirable, and some teen parents carry out their responsibilities exceptionally well. If it were possible to obtain good data on unplanned teen pregnancy or pregnancy by "immature" teen parents, we would use it, but we did not find such data available. Nonetheless, at the aggregate level, it is probably true that public policies or cultural practices that reduce the overall rate of teen births are, other things equal, desirable.

Our findings by themselves, of course, do not permit causal inferences. There could be unstudied confounding variables that account for the correlations we report. But if we may speculate on the most probable explanation, drawing on the other research cited above: we conjecture that conservative religious communities in the U.S. are more successful in discouraging use of contraception among their teen community members than in discouraging sexual intercourse itself.

## Conclusion

At the level of states in the U.S., conservative religious beliefs predict teen birth rates highly and significantly; the correlation remains high and significant after controlling for income and estimated rates of abortion.

## Competing interests

The authors declare that they have no competing interests.

## Authors' contributions

The study was conceived by JMS, who also retrieved the data sets for the analyses. The authors shared the tasks of data entry, organization of the data, statistical analyses, and preparation of the manuscript. Both approved the final manuscript.
